# Effects of Motor Preparation on Walking Ability in Active Ankle Dorsiflexion

**DOI:** 10.3390/neurolint17060093

**Published:** 2025-06-17

**Authors:** Hiroki Ito, Hideaki Yamaguchi, Ryosuke Yamauchi, Ken Kitai, Kazuhei Nishimoto, Takayuki Kodama

**Affiliations:** 1 Graduate School of Health Science, Kyoto Tachibana University, Yamashina-ku 607-8175, Kyoto, Japan; h901524007@st.tachibana-u.ac.jp (H.I.); h901522007@st.tachibana-u.ac.jp (R.Y.); h901523004@st.tachibana-u.ac.jp (K.K.); nishimoto-ka@tachibana-u.ac.jp (K.N.); 2 CARETECH Plus, Nagoya 462-0847, Aichi, Japan; caretech.plus.hy@gmail.com; 3 Cognitive and Molecular Research Institute of Brain Diseases, Kurume University, Kurume 830-0011, Fukuoka, Japan

**Keywords:** motor preparation, active ankle dorsiflexion, voluntary movement, beta power, EEG, corticomuscular coherence (CMC), support-vector machine (SVM), LORETA

## Abstract

Background/Objectives: This study aimed to examine the influence of brain activity during motor preparation on walking ability, focusing on motor control during active ankle dorsiflexion. Methods: Participants were classified into high- and low-corticomuscular coherence (CMC), an index of neuromuscular control based on the median value. Biomechanical and neurophysiological indices of active ankle dorsiflexion and walking ability were compared between the two groups. Additionally, a machine learning model was developed to accurately predict the CMC classification using brain neural activity during motor preparation. Results: The Cz-TA CMC (beta frequency band) during active ankle dorsiflexion successfully detected significant differences in the maximum dorsiflexion angle, inversion angular velocity, brain activity localization, and variations in Cz beta power values during the transition from motor preparation to execution. Furthermore, CMC identified significant differences in dorsiflexion angle changes after toe-off and inversion angles at initial contact during gait. A support-vector machine model predicting high or low CMC demonstrated high accuracy (Accuracy: 0.96, Precision: 0.92–1.00, Recall: 0.91–1.00, F1 Score: 0.95–0.96) during motor execution based on beta power values from −500 to 0 ms prior to the initiation of active ankle dorsiflexion (representing motor preparation). Conclusions: These findings highlight that the motor preparation processes of the brain during active ankle dorsiflexion are involved in walking ability and can be used to predict it. This indicator is independent of disease severity and holds the potential to provide a clinically versatile evaluation method.

## 1. Introduction

Walking is a uniquely acquired bipedal motion that plays a vital role in various aspects of daily life [1]. Walking is essential for functional independence as a means of human mobility [2] and is critical for maintaining the quality of life [3]. Typical clinical evaluation indices of gait performance include gait speed, which is influenced by the gait pattern [4]. Among these, ankle movement control is indispensable for maintaining postural balance and adjusting gait speed through changes in gait patterns [4,5].

Ankle joint control coordinates the timing of ankle joint torque exertion to angular displacement during changes in gait speed [6], and is closely associated with the modulation of gait speed via kinematic alterations in ankle movement [7,8]. However, neurological degeneration due to aging, motor impairments, or other neurological conditions can lead to gait disorders stemming from decreased ankle control [9,10,11]. Improving ankle control is essential for gait rehabilitation, making its evaluation critical for the acquisition or restoration of walking ability. Currently, clinical assessments of ankle control often involve kinematic and kinetic analysis during walking. However, for individuals with severe impairments, direct assessment of walking is often not feasible [12,13]. Therefore, there is a pressing need to identify clinical indicators of ankle control that are independent of the severity and applicable to gait rehabilitation.

From a kinematic perspective, the voluntary dorsiflexion of the ankle is considered important in both the stance and swing phases of gait. Tibialis anterior (TA) muscle activity during dorsiflexion has been shown to resemble cortical motor control observed during walking [14]. Additionally, Dobkin et al. [15] demonstrated via functional magnetic resonance imaging (fMRI) measurements of brain activity that ankle dorsiflexion depends on the descending input from the primary motor cortex and can indirectly assess the spinal sensorimotor network involved in gait control. For these reasons, voluntary dorsiflexion of the ankle under non-weight-bearing conditions has been considered a potentially valuable indicator of lower-limb motor control during gait [16].

However, in clinical practice, there are cases in which the walking ability is impaired despite preserved voluntary dorsiflexion function [17,18], suggesting an inconsistent relationship between the voluntary dorsiflexion function of the ankle and gait ability. Lodha et al. [19] reported that motor control of plantar flexion and dorsiflexion rather than muscle strength may predict walking ability. One method for evaluating motor control is corticomuscular coherence (CMC), which is measured using synchronized electroencephalogram (EEG) and electromyogram (EMG) recordings of the motor areas (Cz) and muscles TA during ankle dorsiflexion [20,21,22]. The β-frequency band of Cz-TA coherence is considered an indicator of neuromuscular control of the lower limbs, with potential links to motor function and walking ability [23].

However, human movement is inherently redundant [24], and existing evaluations lack a comprehensive analysis that integrates both the kinematic and kinetic aspects of movement and the underlying neurophysiological mechanisms. Furthermore, the current evaluation methods rely on actual movements, which may not sufficiently detect foot movements in severely impaired individuals. Therefore, these methods may only be applicable to patients with relatively high levels of voluntary control.

To address this, we drew inspiration from the concept of the motor preparation stage, which involves the simulation of physical movements. Voluntary motor control involves processes such as motor program generation, postural control, and execution of fine movements [25]. Prior to motor execution, motor intention, and planning are processed in the brain during the motor preparation stage [26,27]. Evaluating the entire process from motor preparation to execution may provide insights into the essence of motor control, beyond merely capturing actual movements.

Information processing in the brain during motor preparation may serve as a versatile new indicator of ankle control related to walking ability, even in patients with severe sensorimotor disabilities in whom detecting actual movement is difficult. Furthermore, because this indicator can be measured in the supine, non-weight-bearing position, it has the potential to predict walking ability early and assist in setting clear rehabilitation goals.

The objective of this study was to comprehensively examine the effects of brain information processing during motor preparation for active ankle dorsiflexion on motor control indicators such as CMC and walking ability.

## 2. Materials and Methods

### 2.1. Participants 

The participants of this study were 23 healthy adults, consisting of 18 males and 5 females (age: 22 ± 1 years, height: 1.70 ± 0.06 m, weight: 65.07 ± 14.81 kg). Exclusion criteria included cognitive impairment, sensorimotor dysfunction in the lower limbs, and visual impairment. The study procedures were explained to the participants both orally and in writing, and written informed consent was obtained prior to participation. All participants took part in the experiment after providing informed consent. This study was approved by the Kyoto Tachibana University Research Ethics Committee (Approval Number: 22-13) and conducted in accordance with the Declaration of Helsinki.

### 2.2. Protocol

This study measured ankle joint angles, lower-limb muscle activity, and brain activity during active ankle dorsiflexion and gait tasks. A comprehensive evaluation of ankle control requires a multifaceted approach that includes the assessment of joint angles, muscle activity, and brain activity. Therefore, we utilized the Articulation Motion Assessment System (AMAS) [28], which we developed to enable precise kinematic measurements of joint motion synchronized with other biosignals, such as muscle and brain activity.

The participants wore the AMAS, surface EMG, and scalp EEG devices on one leg. The measurements were performed during two tasks: ankle dorsiflexion and gait.

Active ankle dorsiflexion task

**Figure 1:**
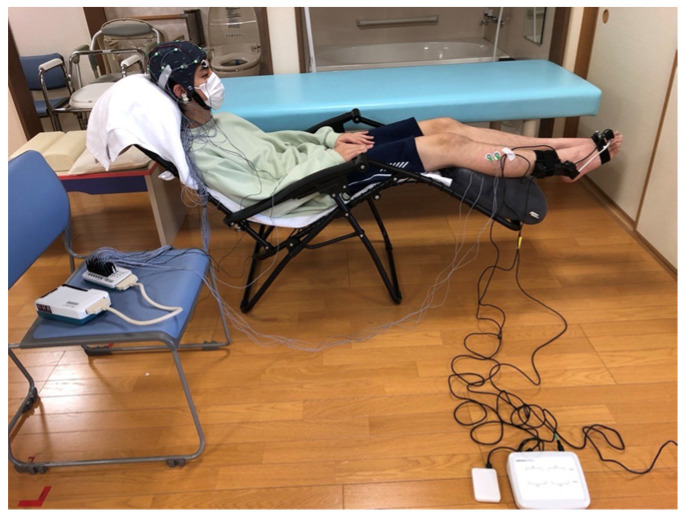
Experimental Setup. Active Ankle Dorsiflexion Task: Participants sat in a chair positioned to allow a clear view of their feet. During the task, a scalp electroencephalogram device and the articulation motion assessment system were carefully set up to prevent entanglement of wires. The angle of the reclining chair was standardized across all participants.

Each participant performed periodic ankle dorsiflexion movements for 3 s, followed by a 7 s rest, and this was repeated 10 times. The tasks were performed at a self-selected pace [23]. Their feet were suspended to allow unrestricted ankle movement (Figure 1).

Walking task

Each participant performed 10 round trips on an 8 m walking path with a 1.5 m acceleration zone at the start and a 1.5 m deceleration zone at the end. A sensor mat was installed on the walking path to detect the initial contact (IC) and toe-off (TO). Participants walked at a comfortable speed.

Regarding ankle movement, previous reports have indicated no significant differences between the left and right sides from both biomechanical and neurophysiological perspectives [29,30]. Therefore, the kicking foot was defined as the dominant foot during the measurements. The right foot was the dominant foot in all participants.

**Figure 2:**
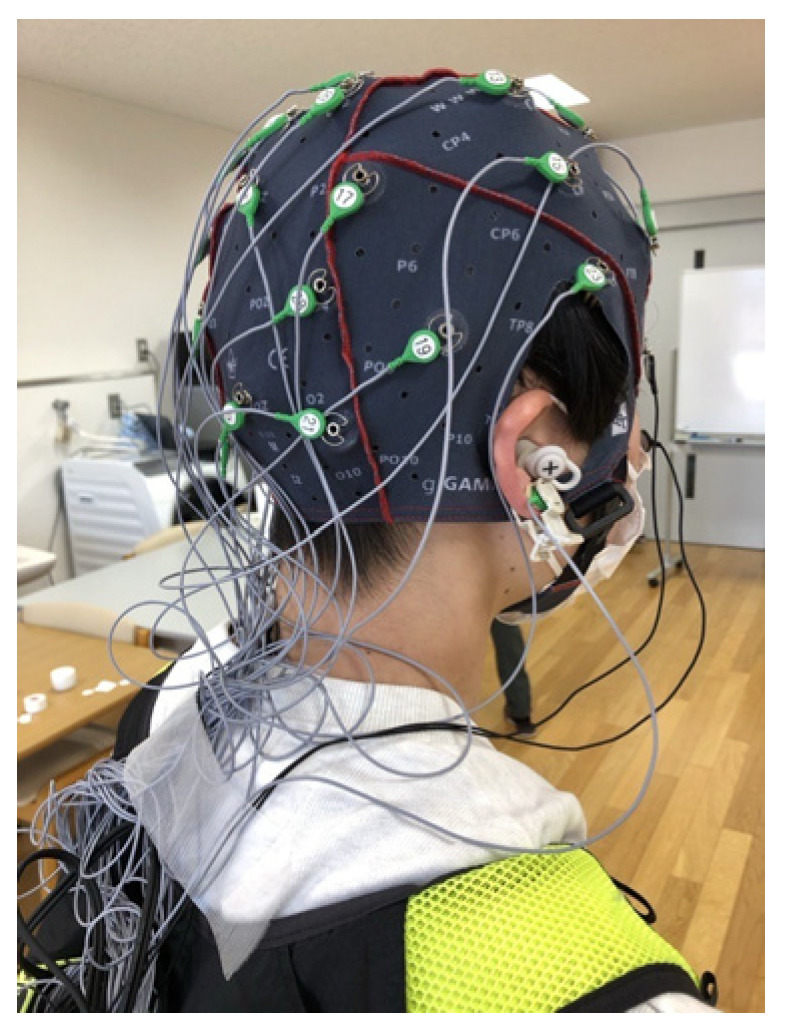
Equipment Setup: Scalp electroencephalogram (EEG). Scalp EEG electrodes were attached using an EEG cap. The wires were loosely bundled to ensure they did not interfere with the movements of the participant. During the gait task, participants carried the scalp EEG device in a small backpack to allow for mobility while walking.

Visual and auditory information processing can also influence motor control [31]. To eliminate these effects, the participants were verbally instructed to look at their feet during the ankle dorsiflexion task and look straight ahead during the walking task to control for visual information processing. In addition, earplugs were used to block external environmental sounds, thereby eliminating the effects of auditory information processing (Figure 2).

### 2.3. Instrumentation

#### 2.3.1. AMAS 

**Figure 3:**
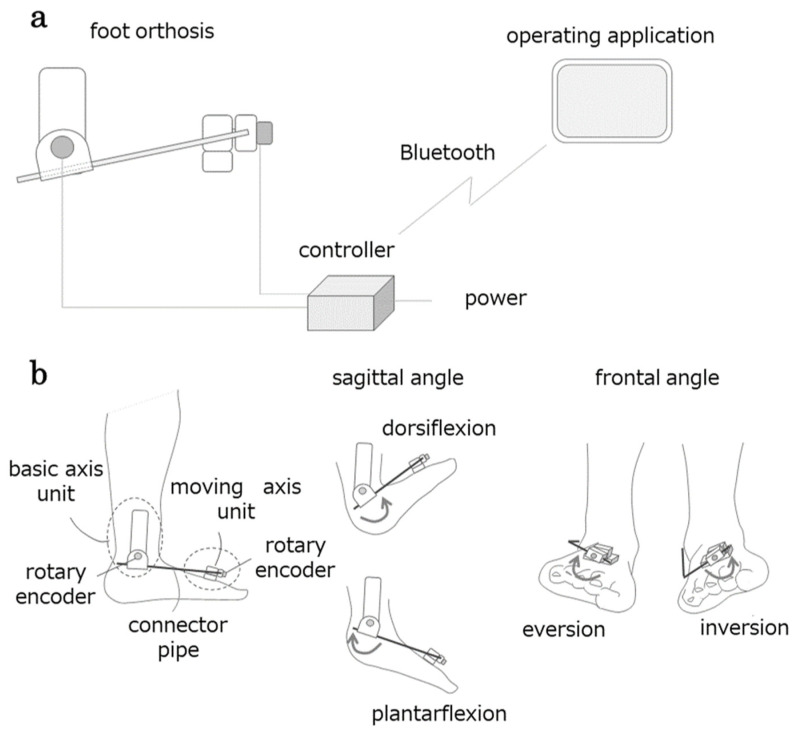
Configuration of the articulation motion assessment system (AMAS). The AMAS comprises three components: a foot orthosis, a controller wirelessly connected to the operating application, and a foot orthosis (**a**). The foot orthosis measures the angles of the sagittal and frontal planes of the foot using a unit incorporating a rotary encoder on the basic axis and moving axis side (**b**).

This joint angle measurement device simultaneously measures the plantar flexion/dorsiflexion and inversion/eversion angles during ankle joint motion and consists of an ankle joint orthosis, controller, and an operation application (Figure 3a). The ankle joint unit (Figure 3b) comprises two units with a built-in rotary encoder (Supertech Electronic, Rixin Street, Chiayi City, Taiwan). One unit was attached to the outer lower leg, which served as the fundamental axis, and the other unit was attached to the dorsal foot (metatarsal head), which served as the moving axis.

A pipe connects both units, and the plantar dorsiflexion movement is transmitted to the basic axis-side unit via the connecting pipe, causing the rotary encoder to rotate. Similarly, the basic shaft-side unit supports the connecting pipe, and the inversion/eversion motion of the ankle is transmitted to the rotary encoder of the mobile shaft-side unit. The controller transmits the signal from the rotary encoder, resulting in ankle motion displayed in the application (tablet). The application converts the signals into angles and displays the changes in plantar flexion/dorsiflexion and inversion/eversion angles in real time. The angle data are saved in a CSV file. The system simultaneously extracts the dorsiflexion/plantar flexion and inversion/eversion angles (degrees) of the ankles. The sampling rate is set to 50 Hz.

#### 2.3.2. Surface Electromyography Measurement

Surface EMG (sEMG) was used to measure muscle activity. The measurement followed a bipolar electrode configuration using Ag-AgCl electrodes (four electrodes), as described in previous studies [23]. Electrode placement was based on the SENIAM guidelines, with an inter-electrode distance of 2 cm. Electrodes were placed on the right TA and medialis gastrocnemius (MG) [32]. For the TA, the electrodes were positioned one-third of the distance along the line connecting the head of the fibula and the tip of the medial malleolus. For the MG, electrodes were placed at the most prominent part of the muscle belly. The sampling frequency was set to 1000 Hz.

#### 2.3.3. EEG Measurement

EEG measurements were conducted using active electrodes and a Polymate V AP5148 biosignal acquisition system (Miyuki Giken Co., Ltd., Tokyo, Japan). The recording electrodes were positioned at 28 sites (FPz, Fz, Cz, Pz, Oz, Fp1, Fp2, F7, F8, F3, F4, C3, C4, C5, C6, P3, P4, P7, P8, O1, O2, T7, T8, CPz, CP1, CP2, PO3, and PO4) according to the international 10–10 system [33]. The ground electrode was placed on the spinous process of the seventh cervical vertebra (C7), and the reference electrode was attached to the right earlobe. In addition, Electro Oculography (EOG) electrodes were placed above and below the eyes to detect eye movement and blinking. The sampling frequency was set to 1000 Hz.

#### 2.3.4. Video Recording and Synchronization

Each task was recorded using two 30-bit/s high-resolution digital video cameras (Panasonic, Tokyo, Japan). For the gait task, cameras were positioned to capture the frontal and sagittal planes, and the time taken to walk along the walkway was recorded. When the participants stepped outside the sensor mat, the IC and TO were visually identified from the recordings. To ensure synchronization, the video cameras and other measurement devices were triggered simultaneously when the participants stepped on the sensor mat.

### 2.4. Data Processing 

**Figure 4:**
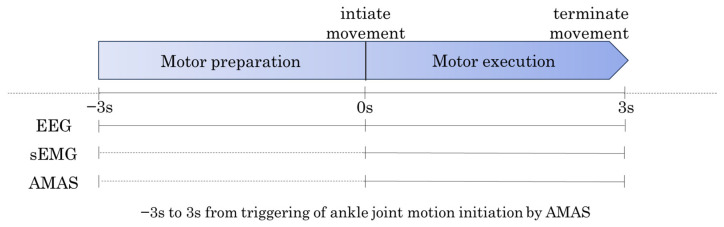
Data Extraction for Ankle Dorsiflexion Task. The data for the ankle dorsiflexion task were divided into two periods. Motor Preparation Period: From 3 s before movement onset (−3 s) to 0 s (movement onset). Motor Execution Period: From 0 s (movement onset) to 3 s after movement onset (+3 s).

The data for the ankle dorsiflexion task were triggered by the start of the ankle movement detected by the AMAS. Each trial consisted of a 6 s period, spanning from 3 s before movement onset to 3 s after movement onset. Data from 10 trials were extracted for the analysis (Figure 4).

#### 2.4.1. Extraction of Biomechanical Indicators

Biomechanical parameters for ankle motion were calculated for 10 repetitions in both the sagittal (dorsiflexion/plantarflexion) and frontal planes (inversion/eversion). The joint angle (°) and angular velocity (rad/s) were computed for each repetition [34,35,36]. The data extraction period was 3 s after the onset of dorsiflexion.

The biomechanical parameters for gait were calculated for 10 gait cycles, focusing on the ankle behavior during IC [37] and TO [38,39]. The joint angle and angular velocity [40,41] were analyzed during these phases. Additionally, gait performance was evaluated by extracting gait speed (m/s) as a performance metric [42,43,44].

The choice of these time windows and parameters was based on prior findings that the functional coupling between cortex and muscle in ankle joint motion is particularly pronounced during these phases [45]. Cortical activity associated with ankle dorsiflexion enhances functional connectivity with TA [46]. Moreover, variations in the ankle joint angles during the stance and swing phases of gait are characteristics of impaired ankle control [47,48,49,50]. These impairments are often accompanied by decreased joint angular velocity, which reflects deficits in muscle activation and force generation dynamics during walking [51]. Data from 10 repetitions of ankle dorsiflexion movements were averaged to obtain representative values.

#### 2.4.2. Preprocessing of sEMG and EEG Data

The sEMG and EEG data were preprocessed using the multimodal EEG analysis program, EMSE Suite (CORETECH SOLUTIONS, Inc., Wilmington, NC, USA; Miyuki Giken Co., Ltd., Japan). First, a band-stop (notch) filter centered at 60 Hz with a bandwidth of 2 Hz was applied to the continuous sEMG and EEG data to remove power line noise from the commercial power supply frequency in western Japan (Kyoto). The sEMG data were filtered using a bandpass filter (5–300 Hz) and were full-wave rectified. The EEG data were filtered using a bandpass filter (0.5–45 Hz).

Artifacts from eye movements and blinking were removed using the EOG artifact removal function implemented in the EMSE.

The preprocessed continuous data were segmented into 6 s intervals, triggered by the onset of ankle dorsiflexion, from 3 s before to 3 s after the movement onset. The same preprocessing was applied to the data from the walking task in which the gait cycle was defined by two consecutive right-foot ICs.

#### 2.4.3. Calculation of CMC 

CMC, a neuromuscular control indicator for lower-limb movements, was calculated as a motor control index for ankle dorsiflexion [52]. The CMC was computed between Cz, TA, and MG. Coherence was derived from the normalization of the cross-spectrum. The formula for coherence is shown below (Equation (1)) [53].

(1) $${Coh}_{xy}\left(f\right)=\frac{{\left|P_{xy}(f)\right|}^{2}}{\left|P_{xx}(f)\right|\cdot \left|P_{yy}(f)\right|}$$

$P_{xy}(f)$: Cross-spectrum between signals x and y.

$P_{xx}(f)$: Power spectral density of signal x.

$P_{yy}(f)$: Power spectral density of signal y.

This metric quantifies the functional connectivity between the motor cortex and target muscles during ankle dorsiflexion movements.

The CMC is a valuable metric for analyzing the functional connectivity of the corticospinal tract between the cerebral cortex and muscles [54]. The coherence values always ranged between zero and one. A value of one indicates that the two signals are in a perfectly coherent (ideal) state. A value of zero indicates that the two signals are completely incoherent. To assess the reliability of the CMC, the confidence level (CL) was calculated using Equation (2) [55]:

(2) $$CL\left(\alpha \right)=1-{\left(1-\alpha \right)}^{\frac{1}{\left(N-1\right)}}$$

N: Represents the number of data segments.

α: Denotes the significance level.

If the calculated CMC value exceeds the 95% significance threshold (α = 0.95), it indicates a significant consistency between cortical and muscle activities. In this study, the total number of data segments was N = 10. This resulted in a CL of 0.275. A CMC value above this threshold suggests statistically significant functional connectivity between the motor cortex and muscles during ankle dorsiflexion.

#### 2.4.4. Calculation of Beta Power Value of EEG by Time-Frequency Analysis

In motor tasks, the event-related desynchronization of β-band power in the motor cortex is associated with motor cortex activation [56,57]. Event-related synchronization is related to post-movement motor evaluation phenomena [58,59]. To reflect the motor control process, fluctuations in β-band power (14–20 Hz), measured in μV^2^, were extracted as an indicator. The region of interest was the motor cortex corresponding to channel Cz. 

A custom MATLAB(R2024b) script was used to perform the time-frequency analysis. This involved convolving a signal with a series of complex Morlet wavelets, which are defined as complex sine waves tapered using a Gaussian function.

The β power fluctuations at channel Cz were expressed as the percentage change in power within the defined time and frequency window relative to a baseline [55]. The baseline period was selected as −3000 to −2500 ms before movement onset.

The analysis used 11 segmented epochs, covering the time period from −2500 ms to +3000 ms around movement onset, as epochs (−2500~−2000 ms/−2000~−1500 ms/−1500~−1000 ms/−1000~−500 ms/−500~0 ms/0~500 ms/500~1000 ms/1000~1500 ms/1500~2000 ms/2000~2500 ms/2500~3000 ms). By setting this time window, the temporal changes in the motor control processes of EEG can be captured.

### 2.5. Statistical Analysis 

All the statistical analyses were performed using SPSS Version 27.0 (IBM Corp., Armonk, NY, USA). The Shapiro–Wilk test was performed to examine the normality of all data. The Cz-TA CMC (β) calculated during ankle dorsiflexion was summarized by its median. Based on this median, the participants were divided into two groups: a CMC-high group (higher CMC values) and a CMC-low group (lower CMC values). To compare the biomechanical indicators (joint angle and joint angular velocity) during active ankle dorsiflexion and walking tasks between the two groups, the following tests were used: independent *t*-tests (for normally distributed data) and the Mann–Whitney U test (for non-normally distributed data) were used. The effect sizes were calculated based on Cohen’s criteria. This analysis aimed to validate whether the Cz-TA CMC (β) during ankle dorsiflexion could differentiate motor control abilities during both active ankle dorsiflexion and walking tasks. To explore the brain’s information processing during motor preparation, the β power values calculated through time-frequency analysis were compared between groups. A significance level of 5% (*p* < 0.05) was set for all the statistical tests.

Standardized low-resolution brain electromagnetic tomography (sLORETA) has been employed to visualize and quantify brain neural activity [60]. Neural activity within brain regions was calculated as the current source density, expressed in μA/mm^2^ × 10^−3^. The regions of activity were identified using Brodmann’s area (BA) and Montreal Neurological Institute (MNI) coordinates [61]. Global Field Power (GFP) values were computed for both groups (CMC-high and CMC-low) using the sLORETA Averager software [60]. The neural activities of the two groups were compared using the sLORETA statistical non-parametric mapping [62]. Brain regions with significant differences in activity (*p* < 0.05) were identified and visually represented using color-coded maps.

A support-vector machine (SVM) model [63] was used to classify the motor control indicators derived from the neural activity during motor preparation [63]. The bootstrap method was applied to resample the original dataset randomly and increase the data size. The number of bootstrap samples varied from 10 to 1000 in increments of 100 to evaluate the effects of data augmentation on the model. The SVM model was trained and tested using a 5-fold cross-validation method. The data are divided into five subsets. Four subsets were used for training. One subset was used for testing. This process was repeated five times to ensure that all subsets were used for testing. To comprehensively evaluate the performance of the model, the following metrics were calculated: Accuracy, Precision, Recall, and F1 Score. Accuracy represents the proportion of correct predictions from all predictions made by the model. The Precision refers to the proportion of true positives among all instances predicted as positive by the model. The Recall measures the proportion of actual positive instances correctly identified by the model. The F1 Score is the harmonic mean of Precision and Recall, balancing the contrasting characteristics. The performance of the model was comprehensively evaluated using these metrics.

## 3. Results

A total of 23 healthy adults participated in this study, with no dropouts. The median Cz-TA CMC (β) value during ankle dorsiflexion was 0.26. The CMC-high group had a mean ± SD of 0.28 ± 0.02 (n = 12), while the CMC-low group had a mean ± SD of 0.23 ± 0.02 (n = 11).

### 3.1. Comparisons of Each Task Parameter Between CMC Groups

#### 3.1.1. Comparison of Biomechanical Indicators in the Active Ankle Dorsiflexion Movement Task

The CMC-high group exhibited significantly greater values than the CMC-low group in dorsiflexion angle change (CMC-high: 34.26 ± 9.25 [°], CMC-low: 25.36 ± 9.43 [°]), peak dorsiflexion angular velocity (CMC-high: 8352.80 ± 3071.86 [rad/s], CMC-low: 5057.30 ± 1345.79 [rad/s]), and inversion angular velocity (CMC-high: 5594.04 ± 2017.03 [rad/s], CMC-low: 3542.88 ± 1159.39 [rad/s]) (*p* < 0.05) (Tables S1 and S2). The effect sizes calculated based on Cohen’s criteria indicated large effects (Cohen’s d = 0.95, 1.37, 1.23).

#### 3.1.2. Comparison of Neurophysiological Indicators in the Active Ankle Dorsiflexion Movement Task

**Figure 5:**
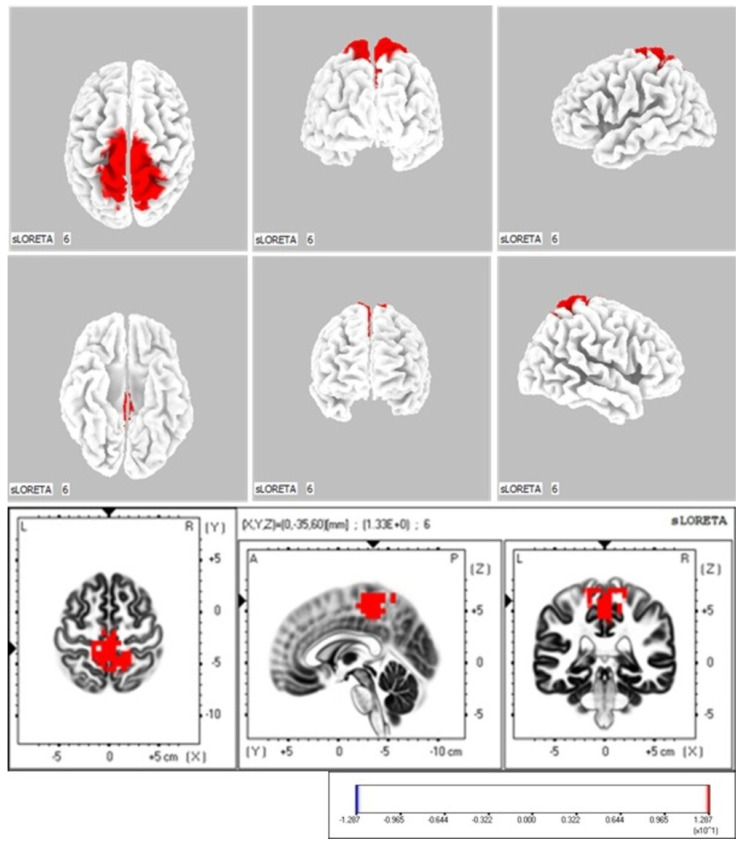
sLORETA Analysis: Localization of Neural Activity During Active Ankle Dorsiflexion Execution The two groups were compared by calculating the difference in global field power (GFP) values between the CMC-high and CMC-low groups. The color-coded neural regions (in red) indicate significant differences, with the GFP values in the CMC-high group being significantly higher than those in the CMC-low group.

The CMC-high and CMC-low groups showed a significant difference in neural activity (*p* < 0.05) in the paracentral lobule (BA 5; MNI coordinates: X = 0, Y = −35, Z = 60) during ankle dorsiflexion (Figure 5).

**Figure 6:**
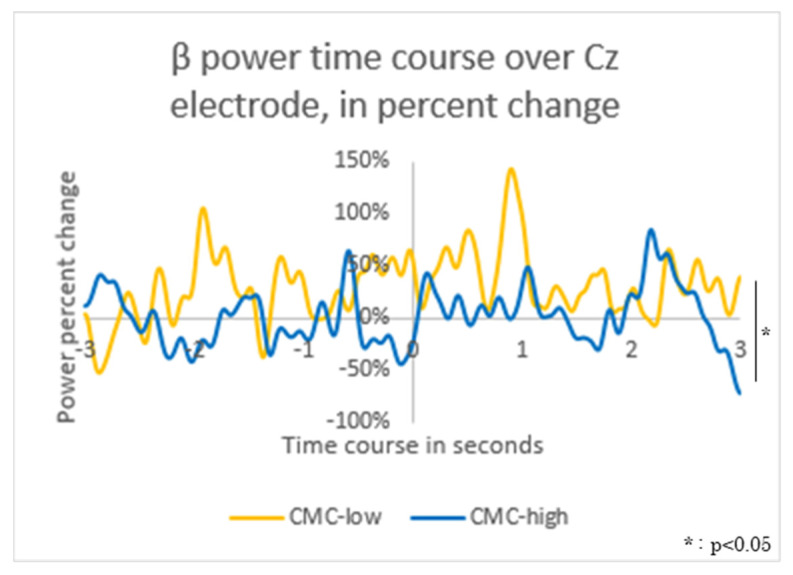
Time-Frequency Analysis: β Power Fluctuations in the Motor Cortex (Cz) The average β power value during −3000 to −2500 ms relative to movement onset was used as the baseline. The fluctuations in β power values for the corticomuscular coherence (CMC)-high group (high: blue solid line) and the CMC-low group (low: yellow solid line) were visualized in a graph.

The β power values at Cz showed a significant difference between the groups (*p* < 0.001) during −0.5 to 0 ms relative to movement onset (CMC-high: −0.40 ± 0.24 [μV^2^], CMC-low: 1.03 ± 0.93 [μV^2^]) and during 0.5 to 1 ms (CMC-high: −0.06 ± 0.70 [μV^2^], CMC-low: 1.12 ± 0.72 [μV^2^]) (Figure 6).

#### 3.1.3. Comparison of Biomechanical Indicators in the Walking Task

During walking, there was no significant difference in walking performance as measured by gait speed between the two groups (CMC-high: 1.20 ± 0.17 [m/s], CMC-low: 1.08 ± 0.16 [m/s]). On the other hand, significant differences were observed in the change in dorsiflexion after TO (CMC-high: 2.23 ± 0.88 [°], CMC-low: 3.51 ± 1.75 [°]) and the inversion angle at IC (CMC-high: 2.51 ± 1.45 [°], CMC-low: 4.72 ± 2.56 [°]) (*p* < 0.05) (Table S3). The effect sizes calculated based on Cohen’s criteria indicated large effects (Cohen’s d = −0.89, −1.00).

### 3.2. Accuracy Assessment of Machine Learning Models (SVM)

The β power fluctuations during motor preparation in ankle dorsiflexion movements demonstrated a high accuracy rate of 0.70 or above in distinguishing high- and low-CMC levels. Notably, the SVM model based on β power fluctuations in the −500 to 0 ms time window achieved an accuracy of 0.96. For Class 1 (CMC-high), the model showed a precision of 1.00, a recall of 0.91, and an F1 Score of 0.95. For Class 2 (CMC-low), the precision was 0.92, the recall was 1.00, and the F1 Score was 0.96. The SVM model based on the −500 to 0 ms β power fluctuations outperformed all other models across all performance evaluation metrics (Table 1).

## 4. Discussion

This study examined the impact of brain information processing during motor preparation in active ankle dorsiflexion on the motor control indicator CMC and walking ability.

In the active ankle dorsiflexion task, the β-band Cz-TA CMC successfully detected significant differences in biomechanical and neurophysiological indicators during motor execution. The β-band CMC serves as a measure of connectivity between the sensorimotor cortex and muscles, influencing changes in the mechanical conditions of movement [64]. Impairment in neuromuscular control resulting from motor dysfunction has been associated with reduced CMC [65]. This suggests that CMC is an indicator of motor control through the connectivity between the cortex and muscles. In this study, CMC values detected differences in biomechanical indicators such as dorsiflexion angles and joint angular velocities in the sagittal and frontal planes during active ankle dorsiflexion. These biomechanical indicators have been clinically used to represent motor control [66,67]. Additionally, CMC values detected differences in neural activity in the paracentral lobule (BA 5; MNI coordinates: X = 0, Y = −35, Z = 60) during ankle dorsiflexion. The paracentral lobule is a cortical region associated with lower-limb movement and is considered a target for rehabilitation interventions for the lower limbs [68]. These findings support the notion that coherence, as functional connectivity between the midline primary motor cortex (Cz) and contracting leg muscles (TA), influences motor execution performance through motor control.

Furthermore, the CMC values during active ankle dorsiflexion detected differences in biomechanical indicators of gait, including the changes in ankle dorsiflexion angle after TO and differences in inversion angle at the IC. Ankle dorsiflexion during TO plays a critical role in preventing foot tripping and falls [36,69]. Reduced dorsiflexion angles and increased variability during the swing phase have been observed [41,70,71]. Changes in these biomechanical indicators contributed to improvements in walking ability [72,73]. Additionally, the angle of the frontal plane ankle at the IC is clinically used as an indicator of functional ankle instability and is associated with the occurrence of ankle sprains [74]. These findings suggest a relationship between motor control during active ankle dorsiflexion and walking ability, highlighting that ankle dorsiflexion influences walking ability not only through muscle strength or joint angle changes but also via motor control aspects, such as neuromuscular connectivity, as previously discussed in related studies [19]. This evaluation method, which can be performed in a non-weight-bearing supine position, provides a quantitative assessment even for individuals who are unable to walk.

However, detection of ankle dorsiflexion movements in clinical practice may not always be feasible. Therefore, this study focused on brain information processing during motor preparation to explore versatile and impairment-independent indicators of ankle control in relation to walking ability. The study revealed that during active ankle dorsiflexion, the CMC values—an indicator of ankle control related to walking ability—clearly detected differences in β-band power fluctuations in the motor cortex (Cz) during the motor preparation period. Voluntary human movement involves processes such as intention, planning, program generation, execution, adjustment, and learning of movements [75]. Neural activity during motor preparation plays a critical role in controlling voluntary movements [76,77], possibly contributing to skilled execution [78,79] and modification [80] of movements during the preparatory state before movement onset, even when no actual movement occurs. Moreover, intentional motor behavior is accompanied by a strong sense of agency arising from predictive brain information processing [81]. These findings suggest a strong link between motor preparation and execution processes in the motor control of ankle dorsiflexion.

Additionally, this study developed a machine learning model to predict the high or low levels of β-band Cz-TA CMC related to walking ability, based on β power during motor preparation in ankle dorsiflexion. The results showed that fluctuations in β power during the −500 to 0 ms period before movement onset most accurately predicted CMC levels during motor execution. Neural activity during motor preparation begins approximately 3 s before movement onset and precedes voluntary movement execution. The magnitude and duration of these fluctuations vary depending on factors such as movement area [82], limb dominance [83], attention to movement timing [84], movement intention [85], and type of movement [86]. Moreover, the significance of these indicators depends on the timing of their occurrence and the neural regions involved [87,88]. The β-band power during motor preparation, emerging from the −500 ms period, is generated by the activity of the sensorimotor cortex [88]. Compared to baseline, β-band power in the sensorimotor cortex decreases by 20–30% during the motor preparation stage [55]. Therefore, the β-band power during this period likely reflects the motor preparation process of voluntary movement. Previous studies have shown that CMC during motor execution reflects both descending and ascending pathways [89], with β-band CMC primarily transmitting descending motor control information [52]. Thus, β-band power during motor preparation for active ankle dorsiflexion is suggested to be involved in neuromuscular control during motor execution related to walking ability and is regulated through descending pathways.

Looking ahead, this indicator holds potential as a clinically versatile evaluation method, allowing the assessment of ankle control through motor intention and planning, even in patients who are unable to perform actual movements.

### Limitations

One limitation of this study was that the participants were healthy young adults without sensorimotor impairments. In this study, high and low levels of CMC, as an indicator of ankle control, did not show a clear difference in gait performance metrics, such as walking speed. Gait speed is influenced by neurological disorders and motor impairment. Therefore, future studies should include participants with gait impairment for further validation. The sample size in this study was relatively small. It can be assumed that increasing the sample size would make the results more generalizable. Therefore, further validation with a larger number of participants is considered necessary in future research.

## 5. Conclusions

This study captured the motor control of active ankle dorsiflexion through multifaceted evaluation as a predictive assessment of walking ability. The β power fluctuations obtained from the Cz EEG electrode, which were the focus of this study, correspond to the simulation of bodily movements that occur before the execution of a movement. This neurophysiological indicator can be detected without the actual movement execution. It identifies the intention to perform a movement and has the potential to become a clinically versatile evaluation metric, independent of impairment severity.

In cases of sensorimotor dysfunction caused by stroke, abnormalities may occur in the neural system that compares motor intentions with the feedback information generated during movement. For such impairments, a neurofeedback training system has been developed and validated to improve the ability to compare intentionally generated movements with actual sensorimotor information [90]. Similarly, EEG signals combined with SVM-based classification have been used to recognize human motor behavior [91,92] and to develop brain–machine interfaces aimed at support and rehabilitation [93,94,95]. The findings of this study suggest potential applications in the field of rehabilitation, not only as an evaluation method for ankle control but also as a foundation for developing feedback systems as an intervention tool. However, this evaluation method may not be applicable to patients with impaired consciousness or cognitive decline who are unable to intend movement. When generalizing this evaluation method to clinical practice, it is necessary to consider the limitations of its applicability.

The multifaceted evaluation of movement using the AMAS, which we developed independently, integrates biomechanical evaluation of movement with neurophysiological evaluation of its underlying mechanisms. The AMAS, which captures the entire process, from movement preparation to execution, is expected to provide new insights into the mechanisms of motor control and contribute to the development of treatments tailored to specific impairments.

## Figures and Tables

**Table 1:** Accuracy of Predictive Models with Support-Vector Machines.

	Accuracy	Precision		Recall		F1 Score	
		High	Low	High	Low	High	Low
−2500~−2000 ms	0.83	0.89	0.79	0.73	0.92	0.80	0.85
−2000~−1500 ms	0.77	0.87	0.72	0.63	0.91	0.73	0.81
−1500~−1000 ms	0.74	1.00	0.67	0.45	1.00	0.62	0.80
−1000~−500 ms	0.70	1.00	0.66	0.25	1.00	0.40	0.80
−500~0 ms	0.96	1.00	0.92	0.91	1.00	0.95	0.96

## Data Availability

The Raw data supporting the conclusions of this study will be made available by the authors upon request.

## Supplementary Materials

Supporting information can be downloaded from https://www.mdpi.com/article/10.3390/neurolint17060093/s1, Table S1. Biomechanical parameters of active ankle dorsiflexion: This table shows the statistics of biomechanical indices in the comparison between the high- and low-CMC groups for active ankle dorsiflexion. Table S2. *β* frequency band CMC of active ankle dorsiflexion movement: This table shows the CMC statistics for the active ankle dorsiflexion movement for the high and low-CMC groups. Table S3. Biomechanical parameters of walking: This table shows the statistics of the biomechanical indices in the high and low-CMC groups for walking.

## Abbreviations

AMAS	Articulation Motion Assessment System
IC	Initial Contact
TO	Toe Off
sEMG	surface electromyography
TA	Tibialis Anterior
MG	Gastrocnemius Medialis
EEG	Electroencephalography
CMC	Corticomuscular Coherence
sLORETA	Standardized Low-Resolution Brain Electromagnetic Tomography
BA	Brodmann’s area
GFP	global field power
SVM	Support-Vector Machine
