# Supplementary material for: Effects of Motor Preparation on Walking Ability in Active Ankle Dorsiflexion
Source: Neurol Int. 2025 Jun 17;17(6):93. doi: 10.3390/neurolint17060093 (PMC12196276; doi:10.3390/neurolint17060093)
Supplement: Supplementary file 1 [file neurolint-17-00093-s001.zip › Table S1.pdf]

Table S1. Biomechanical parameters of active ankle dorsiflexion movement

Parameter	group	Mean	SD	SE	
Initial ankle joint angle (sagittal plane)[°]	high	-19.86	10.09	2.91	
	low	-16.41	12.07	3.64	
Maximum dorsiflexion angle[°]	high	14.39	11.67	3.37	
	low	8.95	12.94	3.90	
Change in dorsiflexion angle [°]	high	34.26	9.25	2.67	*
	low	25.36	9.43	2.84	
Maximum dorsiflexion angular velocity[rad/s]	high	8352.80	3071.86	886.77	*
	low	5057.30	1345.79	405.77	
Initial ankle joint (frontal plane)[°]	high	-26.15	18.59	5.37	
	low	-27.31	21.61	6.51	
Maximum inversion angle[°]	high	-5.76	22.97	6.63	
	low	-15.51	22.97	6.93	
Change in inversion angle[°]	high	20.48	12.56	3.63	
	low	11.87	7.96	2.40	

Maximum inversion angular velocity[rad/s]	high	5594.04	2017.03	582.27	*
	low	3542.88	1159.39	349.57	

* : p<0.05
