# Supplementary material for: Effects of Motor Preparation on Walking Ability in Active Ankle Dorsiflexion
Source: Neurol Int. 2025 Jun 17;17(6):93. doi: 10.3390/neurolint17060093 (PMC12196276; doi:10.3390/neurolint17060093)
Supplement: Supplementary file 1 [file neurolint-17-00093-s001.zip › Table S2.pdf]

Table S2. β frequency band CMC of active ankle dorsiflexion movement

Parameter	group	Mean	SD	SE	
Cz-TA	high	0.28	0.02	0.00	*
	low	0.23	0.02	0.00	
Cz-MG	high	0.26	0.04	0.00	
	low	0.26	0.04	0.00	
TA-MG	high	0.31	0.05	0.00	
	low	0.28	0.04	0.00	

* : $p<0.05$
