# Supplementary material for: Effects of Motor Preparation on Walking Ability in Active Ankle Dorsiflexion
Source: Neurol Int. 2025 Jun 17;17(6):93. doi: 10.3390/neurolint17060093 (PMC12196276; doi:10.3390/neurolint17060093)
Supplement: Supplementary file 1 [file neurolint-17-00093-s001.zip › Table S3.pdf]

Table S3. Biomechanical parameters of walking

Parameter	group	Mean	SD	SE	
IC Dorsiflexion angle[°]	high	2.89	1.33	0.38	
	low	2.90	1.18	0.35	
Amount of dorsiflexion change after TO[°]	high	2.23	0.92	0.27	*
	low	3.51	1.84	0.55	
Maximum dorsiflexion angular velocity after TO[rad/s]	high	2415.68	645.03	186.20	
	low	4359.76	3297.45	994.22	
TO Dorsiflexion angle[°]	high	8.24	2.06	0.59	
	low	8.55	2.10	0.63	
IC Inversion angle[°]	high	2.51	1.52	0.44	*
	low	4.71	2.68	0.81	
Maximum inversion angular velocity after TO[rad/s]	high	3591.70	1559.61	450.22	
	low	3093.78	1524.59	459.68	
Amount of inversion change after TO[°]	high	2.80	1.19	0.34	
	low	2.46	2.08	0.63	
TO Inversion angle[°]	high	5.06	3.02	0.87	

	low	5.80	2.87	0.86
Gait speed[m/s]	high	1.20	0.17	0.05
	low	1.08	0.16	0.05

* : p<0.05
